# AST-120 alleviates renal ischemia-reperfusion injury by inhibiting HK2-mediated glycolysis

**DOI:** 10.1186/s10020-024-00902-y

**Published:** 2024-08-31

**Authors:** Jinmeng Zhou, Jinbao Zhang, Feng Xu, Haijin Gao, Lei Wang, Yutong Zhao, Ke Li

**Affiliations:** Department of Critical Care Medicine, the First Hospital of Shanxi Medical University, 85 Jiefang South Road, Yingze District, Taiyuan, 030001 Shanxi China

**Keywords:** Renal ischemia-reperfusion injury, AST-120, Glycolysis, HK2, Histone lactylation

## Abstract

**Objective:**

Renal ischemia/reperfusion injury (IRI) is a major cause of acute kidney injury (AKI), which is associated with high incidence and mortality. AST-120 is an oral carbonaceous adsorbent that can alleviate kidney damage. This study aimed to explore the effects of AST-120 on renal IRI and the molecular mechanism.

**Methods:**

A renal IRI mouse model was established and administrated AST-120, and differentially expressed genes were screened using RNA sequencing. Renal function and pathology were analyzed in mice. Hypoxia/reoxygenation (H/R) cell model was generated, and glycolysis was evaluated by detecting lactate levels and Seahorse analysis. Histone lactylation was analyzed by western blotting, and its relationship with hexokinase 2 (HK2) was assessed using chromatin immunoprecipitation.

**Results:**

The results showed that HK2 expression was increased after IRI, and AST-120 decreased HK2 expression. Knockout of HK2 attenuated renal IRI and inhibits glycolysis. AST-120 inhibited renal IRI in the presence of HK2 rather than HK2 absence. In proximal tubular cells, knockdown of HK2 suppressed glycolysis and H3K18 lactylation caused by H/R. H3K18 lactylation was enriched in HK2 promoter and upregulated HK2 levels. Rescue experiments revealed that lactate reversed IRI that suppressed by HK2 knockdown.

**Conclusions:**

In conclusion, AST-120 alleviates renal IRI via suppressing HK2-mediated glycolysis, which suppresses H3K18 lactylation and further reduces HK2 levels. This study proposes a novel mechanism by which AST-120 alleviates IRI.

## Introduction

Acute kidney injury (AKI) refers to a sudden loss of kidney function clinical heterogeneous syndrome, mostly occurring during infection, trauma, and surgery [1, 2]. It affects approximately 50% of critically ill patients, leading to high in-hospital mortality and long-term poor prognosis [3]. Renal ischemia/reperfusion injury (IRI) is a major reason for AKI [4]. Reperfusion therapy is the standard option for restoring blood supply to ischemic tissue and may contribute to improving ischemic injury. However, even if blood supply is restored, the function of the kidney may not be restored but is further aggravated [5]. IRI leads to abnormal apoptosis of renal tubular cells and inflammation, which are the pathological mechanisms of AKI development [6]. Therefore, alleviating renal IRI may be critical in developing novel treatments for AKI.

Studies have reported that AST-120 (KREMEZIN) is approved to treat chronic kidney diseases (CKD) [7, 8]. AST-120 is an oral spherical carbonaceous adsorbent that can adsorb indole to inhibit the production of indoxyl sulfate, which is thought to promote CKD progress. Patients with CKD who received AST-120 treatment have delayed initiation of dialysis, and the mortality and stroke incidence are reduced [9]. Several studies have shown that oral administration of AST-120 possesses the function of alleviating renal IRI, and thereby impeding AKI development [10, 11]. However, the underlying mechanism of AST-120 function in renal IRI is complex and has not been well documented.

Glycolysis is a metabolism process that oxidizes pyruvate to acetyl-CoA under aerobic conditions and reduces pyruvate to lactic acid under anaerobic conditions, generating ATP [12]. Abnormal glycolysis is involved in the progression of numerous diseases, such as atherosclerosis, malignancy, and inflammation [13–15]. In AKI, proximal tubular cells increase glycolysis to prevent kidney damage against stress in the short term, but still lead to renal injury in the long term [16]. When IRI occurs, a decrease in local blood flow leads to hypoxia, and the kidneys exhibit an increase in lactic acid and pyruvate content, suggesting the promotion of glycolysis [17]. Hexokinase 2 (HK2), a rate-limiting enzyme in glycolysis, catalyzes glucose phosphorylation to glucose 6-phosphate (G6P) [18]. Therefore, it is essential to examine the role of HK2-mediated glycolysis in renal IRI.

Anaerobic glycolysis describes the production of lactate, which derives the lactylation modification of histone lysine residues. As a protein post-translation modification (PTM), it regulates gene transcription from chromatin [19]. Histone lactylation marks lactate levels, suggesting that it may be a switch for glycolysis [20]. Growing evidence has revealed that histone lactylation regulates several biological activities and drives disease progress, such as cancer, inflammation, neuropathy, and fibrotic disease [21]. However, the role of histone lactylation in AKI, especially the effects on renal IRI, remains not understood.

In this study, we aimed to identify the effects of AST-120 on renal IRI and its molecular mechanisms. We speculated that AST-120 affected IRI by regulating HK2-mediated glycolysis, and that histone lactation is involved in this process. The study may provide a novel mechanism for AST-120 to regulate renal IRI.

## Materials and methods

### Animal study

The animal procedures were approved by the Ethics Committee of MDKN Biotechnology Co., Lt. Wild-type (WT) and HK2 knockout (KO) C57BL/6 mice (male, 10–12 weeks old) were obtained from Cyagen (Suzhou, China). All mice were housed in specific pathogen free conditions under 12/12 h light/dark cycles with food and water. WT or HK2 KO mice were divided into sham, I/R, and AST-120 groups (6 mice per group). To establish I/R mice, mice were anesthetized with intraperitoneal injection (i.p.) of 50 mg/kg sodium pentobarbital. After the lateral ventral incision, bilateral renal pedicles were exposed for clamping for 30 min to induce ischemia. Then, the clamps were removed for 48 h for reperfusion. The sham mice underwent the same surgery without clamping. During the surgery, the mice were kept on a heated blanket at 37 °C. To explore the role of AST-120 in vivo, the mice were orally administrated 125 µg/kg AST-120 (Kureha Corporation, Tokyo, China) for 2 days, twice a day [11]. Finally, the mice were sacrificed by overdose of sodium pentobarbital (i.p.). The blood samples and renal tissues were collected from all mice for further study.

### RNA sequencing (RNA-seq)

Total RNA was isolated from renal tissues of WT mice in the sham, I/R, and AST-120 groups using the RNeasy Mini kit (Qiagen, Duesseldorf, Germany). RNA quality was measured using a NanoDrop 2000 (Thermo Fisher Scientific, Waltham, USA). RNA integrity was evaluated using agarose gel electrophoresis. RNA-seq library was constructed using the SMARTer Stranded RNA-Seq kit (Clontech, Mountain View, CA, USA). RNA was reversed transcribed to synthesize cDNA first and second chains. cDNA library sequencing was performed on a HiSeq 4000 instrument (Illumina, San Diego, CA, USA).

### Determination of renal function

The blood was centrifuged at 2500 rpm for 10 min to collect serum. The concentrations of serum creatinine (SCr) and blood urea nitrogen (BUN) were measured in serum using the creatinine assay kit (Abcam, Cambridge, MA, USA) and BUN colorimetric detection kit (nwbiotec, Beijing, China) according to the manufacturer’s instructions, respectively.

### Isolation of platelets

The blood was collected in blood collection tubes, and EDTA was added as the anticoagulant. The isolation of platelets was performed as previously described [22]. The blood was centrifuged at 300 g for 10 min at room temperature to obtain platelet-rich plasma. Next, the sample was further centrifuged at 1600 g for 10 min, and platelets were collected. The pellets were suspended in Dulbecco’s modified Eagle’s medium (DMEM; Gibco, Grand Island, NY, USA) supplemented with pyruvate, EDTA, D-glucose, and L-glutamine.

### Histopathological examination

Renal pathology was visualized using hematoxylin and eosin (H&E) staining assay. Renal tissues were fixed with 4% paraformaldehyde, dehydrated using graded ethanol, and hyalinized using xylene. Then, the tissues were embedded in paraffin, and the paraffin Sect. (5 μm) were made. After deparaffined and rehydrated, the sections were stained with hematoxylin and eosin (Sigma-Aldrich, St. Louis, MO, USA), and the staining results were imaged using a microscope. Renal tubular injury was evaluated by two investigators who were blinded to the experiments. Ten nonoverlapping fields were randomly selected to assessed to injury score. Kidney injury score was evaluated based on the percentage of damaged tubules per field: 0 point, normal; 1 point, < 5%; 2 points, 5–25%; 3 points, 26–75%; 4 points, > 75%).

### TUNEL assay

A TUNEL kit (Elabscience, Wuhan, China) was used to analyze apoptosis in renal tissues. The paraffin sections of tissues were deparaffined, rehydrated, and incubated with Protease K at 37 °C for 20 min. After washing with PBS, 100 µL TdT Equilibration buffer was added to each sample to incubate for 30 min. Then, 50 µL labeling solution was incubated with the sections for 1 h. The sections were further washed with PBS and incubated with DAPI solution for 5 min in the dark. Apoptosis was observed under a fluorescence microscope. TUNEL positive cells were quantified.

### Cell culture and stimulation

Human proximal tubular cell line HK-2 was purchased from ATCC (Manassas, VA, USA). The cells were cultured in DMEM supplemented with 10% fetal bovine serum (FBS; Gibco) at 37 °C with 5% CO_2_.

To generate a hypoxia/reoxygenation (H/R) cell model, HK-2 cells were cultured in hypoxia conditions (94% N_2_, 1% O_2_, and 5% CO_2_) for 24 h and normal oxygen conditions (74% N_2_, 21% O_2_, and 5% CO_2_) for 12 h as previously described [23].

To promote glycolysis, HK-2 cells were exposed to 10 mM lactate (LA; Sigma-Aldrich) for 24 h. To inhibit glycolysis, 10 mM 2-Deoxy-d-glucose (2-DG; Sigma-Aldrich) was used to treat HK-2 cells for 24 h.

### Cell transfection

Short hairpin RNA targeting HK2 (sh-HK2), the negative control (sh-NC), HK-2 overexpression plasmid, and empty vector were designed and synthesized in GenePharma (Shanghai, China). They were transfected into HK-2 cells in six-well plates using Lipofectamine 2000 (Invitrogen, Carlsbad, CA, USA). After 48 h, the transfected cells were collected.

### Determination of lactate level

The lactate concentration in renal tissues and HK-2 cells was measured using the L-Lactic Acid colorimetric assay kit (Elabscience) following the manufacturer’s protocol. The absorbance was detected at 530 nm using a microplate reader.

### Cell counting kit-8 (CCK-8)

HK-2 cells (100 µL, 2 × 10^4^ cells/mL) were seeded in 96-well plates and pre-cultured for 24 h. Then, 10 µL CCK-8 reagent from a CCK-8 kit (Abmole, Shanghai, China) was added to the plates to incubate with the cells for 4 h. The absorbance was measured at 450 nm using a microplate reader.

### Assessment of extracellular acidification rate (ECAR) and oxygen consumption rate (OCR)

HK-2 cells were seeded into XF96 culture plates. ECAR and OCR were measured using the Seahorse XF96 extracellular flux analyzer (Agilent Technologies, Santa Clara, CA, USA). Glucose, oligomycin A, and 2-DG were sequentially added automatically for ECAR analysis. Oligomycin A, carbonyl cyanide 4-(trifluoromethoxy)phenylhydrazone (FCCP), and antimycin A and rotenone (Rote/AA) were sequentially added automatically for OCR analysis. Data were analyzed using the Seahorse Wave Desktop software.

### Quantitative real-time polymerase chain reaction (qPCR)

Total RNA was extracted from HK-2 cells using TRIzol reagent (Invitrogen). Next, RNA was reversed transcribed to cDNA using the Hifair^®^ AdvanceFast 1st Strand cDNA Synthesis Kit (Yeasen, Shanghai, China). Then, Hieff^®^ qPCR SYBR Green Master Mix (No Rox) (Yeasen) was used to conduct qPCR on the CFX96 instrument (Bio-Rad, Hercules, CA, USA). Expression was calculated using the 2^−ΔΔCt^ method by normalizing to the internal control, GAPDH.

### Immunoprecipitation (IP)

HK-2 cells were lysed using the IP buffer. After centrifuging at 13,000 g for 10 min, the supernatant was collected and incubated with anti-HK2 and Dynabeads magnetic beads (Thermo Fisher Scientific) at 4 °C overnight. Then, the magnetic beads were washed to remove unbound proteins. After eluting the proteins, western blotting was performed.

### Western blotting

Renal tissues and HK-2 cells were lysed using the radio-immunoprecipitation assay (RIPA) buffer (Beyotime). The isolated proteins (about 30 µg each lane) were separated using 10% SDS-PAGE, and then transferred onto polyvinylidene fluoride membranes. The membranes were blocked using the Fast Blocking Western reagent (Yeasen), incubated with primary antibodies for one night at 4 °C, followed by the incubation of secondary antibodies for 1 h. Each immunoreactive protein band was visualized using the Super ECL detection reagent (Yeasen). The results were quantified using the ImageJ software.

### Chromatin immunoprecipitation (CHIP)

HK-2 cells were cross-linked with 1% methanal at 37 °C for 10 min, and glycine was added to stop the cross-link. The cells were collected in SDS buffer (10^6^ cells/100 µL) and were broken ultrasonically. The cells were incubated with antibodies (IgG and H3K18la) and Dynabeads magnetic beads at 4 °C overnight. After washing in low salt, high salt, LiCl, and TE wash buffer, DNA-protein bound fractions were eluted. Cross-link was reversed at 65 °C overnight. DNA sample was obtained, and qPCR analyzed NK2 promoter region expression.

### Statistical analysis

All in vitro experiments were independently repeated three times. The in vivo data were acquired from six mice in each group. Data are presented as the mean ± SD. Student’s t test or one-way ANOVA was used to compare differences for two or multiple groups. p-value < 0.05 was considered statistically significant.

## Results

AST-120 affects HK2 expression after renal IRI.

**Fig. 1:**
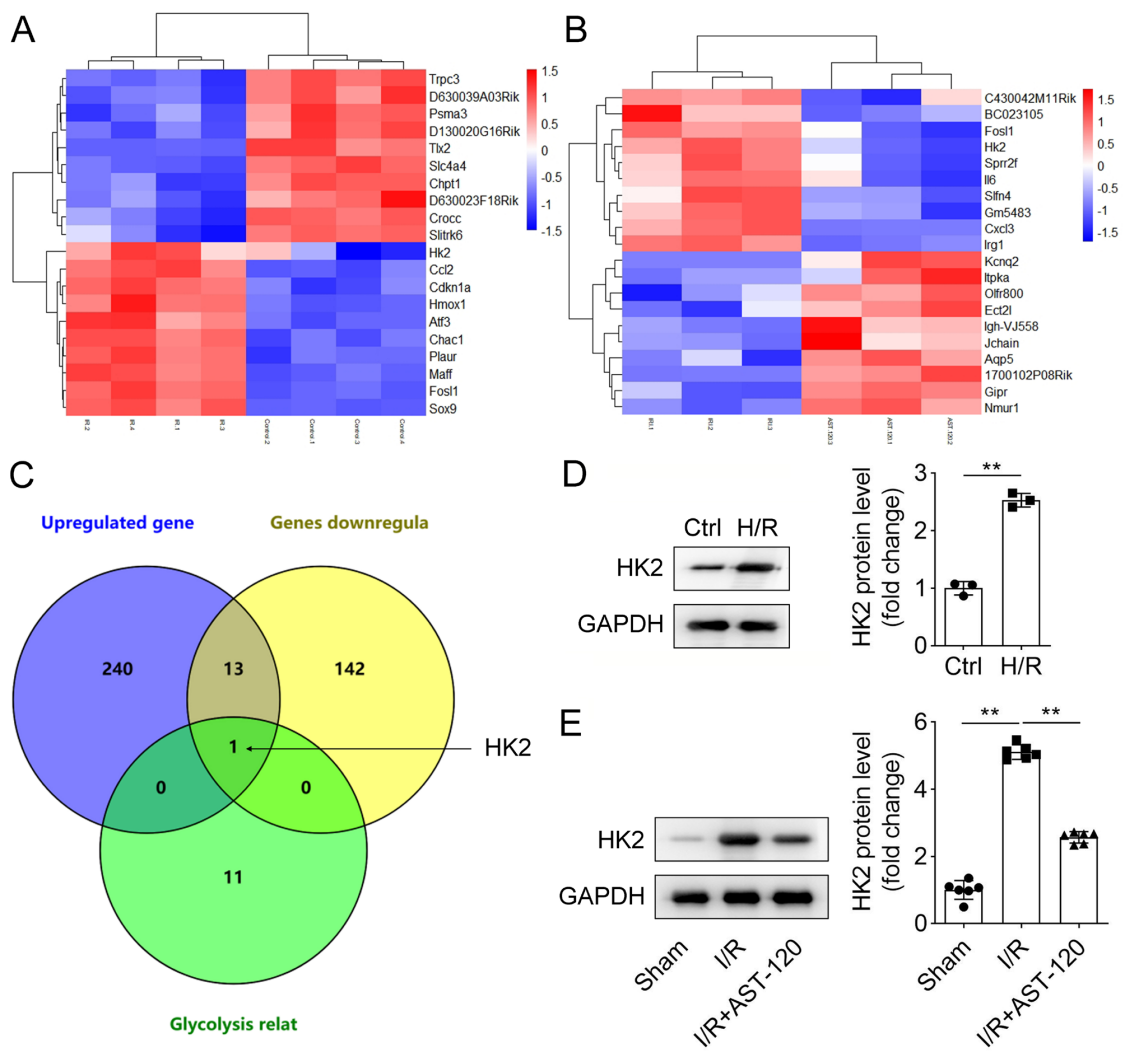
AST-120 affects HK2 expression after renal IRI. (**A**) Differentially expressed genes (top 10 upregulated and 10 downregulated) in kidney tissues from sham and I/R mice were predicted using RNA-seq. (**B**) Differentially expressed genes (top 10 upregulated and 10 downregulated) in renal tissues of I/R mice treated with or without AST-120. (**C**) Upregulated genes in I/R mice, downregulated genes caused by AST-120, and glycolysis-related genes are shown using the Venn diagram. (**D**) HK2 protein levels were detected in HK-2 cells treated with or without H/R using western blotting. (**E**) Western blotting was performed to examine HK2 levels in I/R mice treated with or without AST-120

To explore which gene is associated with IRI, we established the I/R mouse model, and the sham mice were the control. RNA-seq was performed to analyze differentially expressed genes. A total of 254 upregulated and 62 downregulated genes were found in IRI mice. The top 10 differentially expressed genes are shown in Fig. 1A. Subsequently, IR mice were treated with AST-120, and the differentially expressed genes were also assessed using RNA-seq. The results indicated that 21 genes were upregulated and 156 genes were downregulated after AST-120 treatment. The top 10 differentially expressed genes are shown in Fig. 1B. Renal IRI prevents oxygen levels in the kidney from returning to normal [24], and the lack of oxygen induces glycolysis. Thus, we would like to evaluate whether glycolytic-related enzymes are involved in IRI. As shown in the Venn diagram, only HK2, a glycolytic rate-limiting enzyme, was upregulated in IR mice and downregulated after AST-120 treatment (Fig. 1C). Thus, we chose HK2 for further study. Its protein levels were detected in cells and mice. HK2 protein levels were highly expressed in H/R-induced cells (Fig. 1D). Moreover, protein levels of HK2 were upregulated in I/R mice, and AST-120 reduced its levels in I/R mice (Fig. 1E). The results demonstrated that IRI increases HK2 expression, which is reduced by AST-120.

### Knockout of HK2 alleviates kidney IRI in mice

**Fig. 2:**
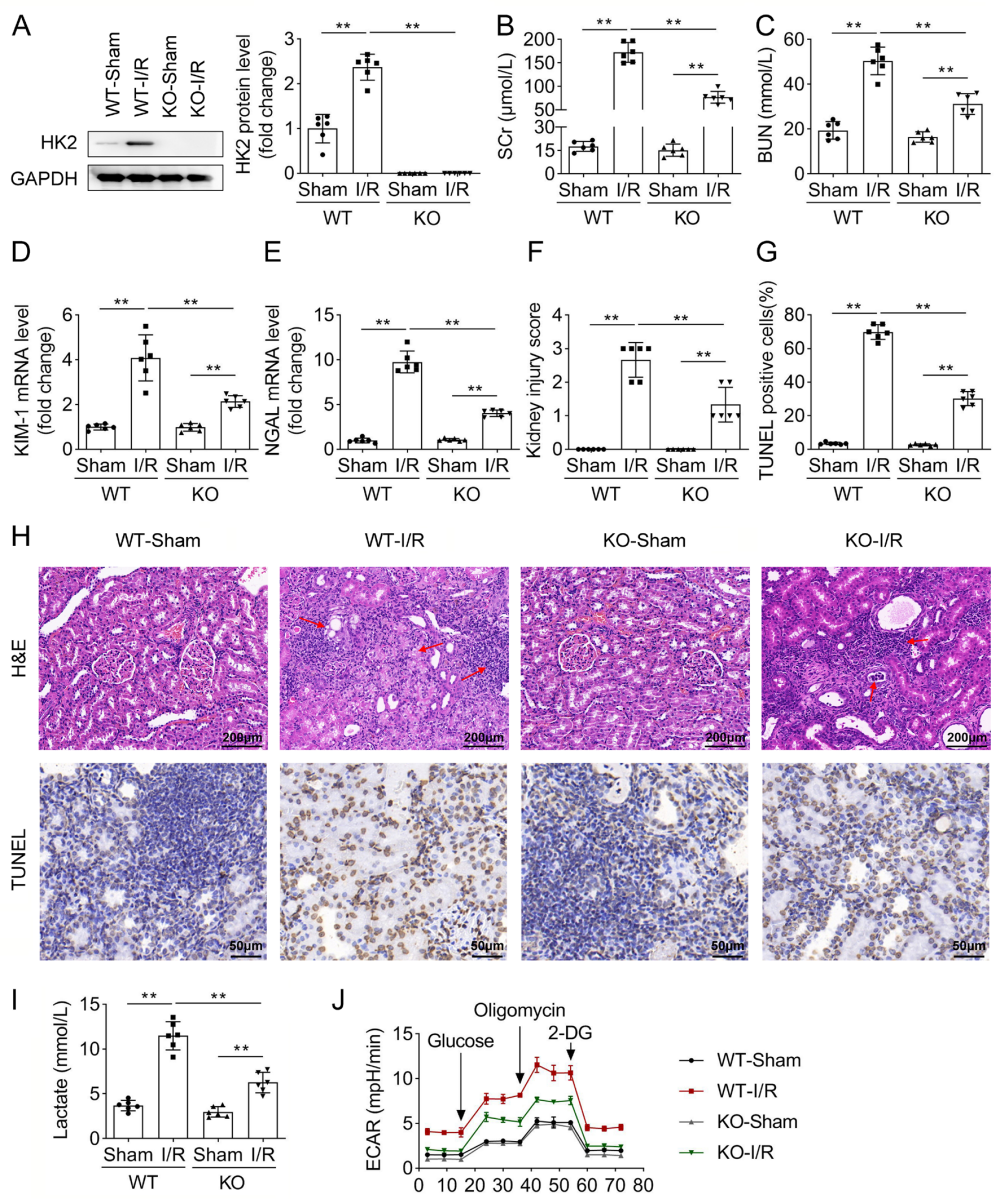
Knockout of HK2 alleviates kidney IRI in mice. (**A**) Protein levels of HK2 were examined using western blotting in WT or HK2-KO mice in the sham and I/R groups. (**B**) SCr and (**C**) BUN levels in the serum of mice were measured using corresponding commercial kits. (**D**) KIM-1 and (**E**) NGAL expression in the renal tissues of mice were measured using qPCR. (**F**) Kidney injury score according to the H&E staining. (**G**) The percentage of TUNEL positive cells was quantified. (H) Representative images of H&E staining (scale bar: 200 μm; red arrows: injured tissue) and TUNEL assays (scale bar: 50 μm). (**I**) Lactate levels were measured using a corresponding kit. (J) Platelets were isolated from the blood of mice, and ECAR was analyzed using a Seahorse assay. **P < 0.01

As mentioned above, HK2 is predicted to be highly expressed in I/R mice. Therefore, whether HK2 is involved in renal injury needs to further confirmed. We used WT and HK2 KO mice to establish the I/R mouse model. In WT mice, HK2 levels were elevated after the I/R model was established and were deleted in HK2-KO mice in the sham and I/R groups (Fig. 2A). Next, we evaluated renal functions. After I/R, SCr and BUN levels were both increased in WT and HK2-KO mice. As compared to WT mice in the I/R group, SCr and BUN levels were low in HK2-KO mice in the I/R group (Fig. 2B and C). The mRNA expression of KIM-1 and NGAL, which are proximal tubule injury markers [25], were measured in the renal tissues. I/R elevated KIM-1 and NGAL expression in both WT and HK2-KO mice. Knockout of HK2 decreased their levels, compared with the WT group (Fig. 2D and E). Later, H&E assay was performed to analyze histopathological damage. The results showed that I/R increased pathological injury of kidney tissues, and knockout of HK2 reduced the injury (Fig. 2F and H). Besides, TUNEL assay was used to evaluate cell death. Cell death was increased in I/R mice, whereas HK2 KO reduced cell death caused by I/R (Fig. 2G and H). Lactate is the end-product of glycolysis. Thus, we analyzed the lactate levels in renal tissues. I/R increased lactate levels, whereas knockout of HK2 reversed lactate levels in I/R mice (Fig. 2I). Additionally, renal IRI activates platelets, further promoting inflammation and coagulation [26]. Glycolysis is essential for ATP production from platelets. Hence, ECAR in platelets was examined. ECAR in platelets was increased by I/R induction in both WT and HK2 KO mice, and the increase was more significant in the WT mice (Fig. 2J). Taken together, I/R causes renal damage in vivo, and deficiency of HK2 attenuated renal IRI.

### AST-120 attenuates renal injury in I/R mice by regulating HK2

**Fig. 3:**
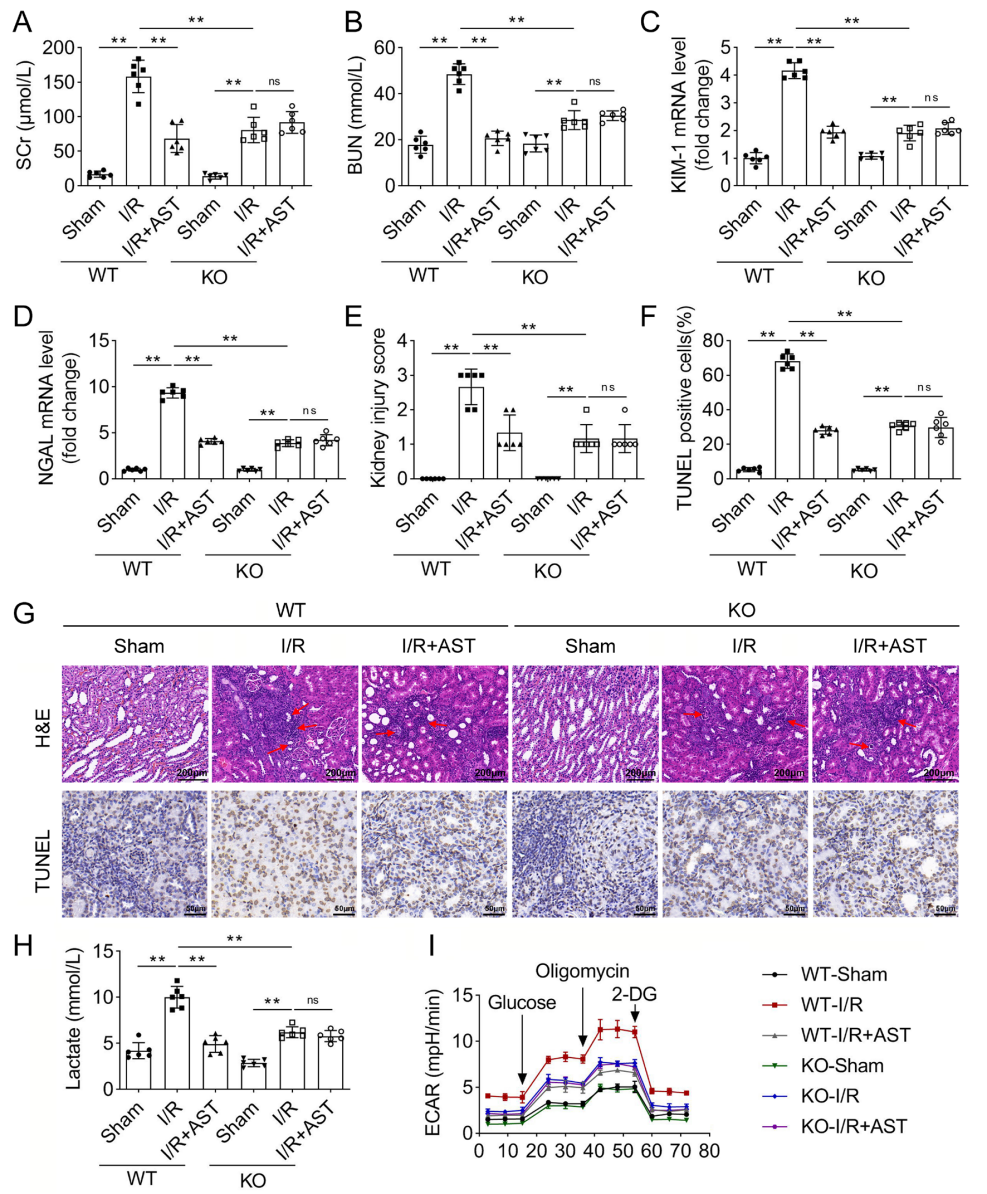
AST-120 attenuates renal injury in I/R mice by regulating HK2. (**A**) SCr and (**B**) BUN levels were measured in the serum of mice using commercial kits. (**C**) KIM-1 and (**D**) NGAL expression in the renal tissues of mice were measured using qPCR. (**E**) Kidney injury score according to the H&E staining results. (**F**) The percentage of TUNEL positive cells. (**G**) Representative images of H&E staining (scale bar: 200 μm; red arrows: injured tissue) and TUNEL assays (scale bar: 50 μm). (**H**) Lactate levels were measured. (**I**) ECAR in platelets of mice was measured using a Seahorse analysis. **P < 0.01. ns, no significant

Subsequently, we analyzed the role of AST-120 in vivo. I/R mice were administrated AST-120. Then, indicators of renal function and renal injury were measured. We found that in the WT group, AST-120 reduced SCr and BUN levels; however, AST-120 did not affect their levels in HK2-KO mice (Fig. 3A and B). The expression of KIM-1 and NGAL was downregulated by AST-120 in I/R WT mice, whereas AST-120 did not regulate their expression in I/R HK2-KO mice (Fig. 3C and D). In addition, AST-120 improved I/R-induced renal tissue injury and cell death in WT mice, but failed to affect renal injury in HK2-KO mice (Fig. 3E-G). Moreover, lactate levels that increased by I/R were reversed by AST-120 in WT mice rather than HK2-KO mice (Fig. 3H). Platelet ECAR was increased by I/R treatment in WT and HK2-KO mice. AST-120 administration counteracted ECAR in WT mice, but did not affect ECAR in HK2-KO mice (Fig. 3I). Collectively, AST-120 attenuates renal IRI in mice in the presence of HK2.

### Silencing of HK2 suppresses glycolysis in H/R-induced HK-2 cells

**Fig. 4:**
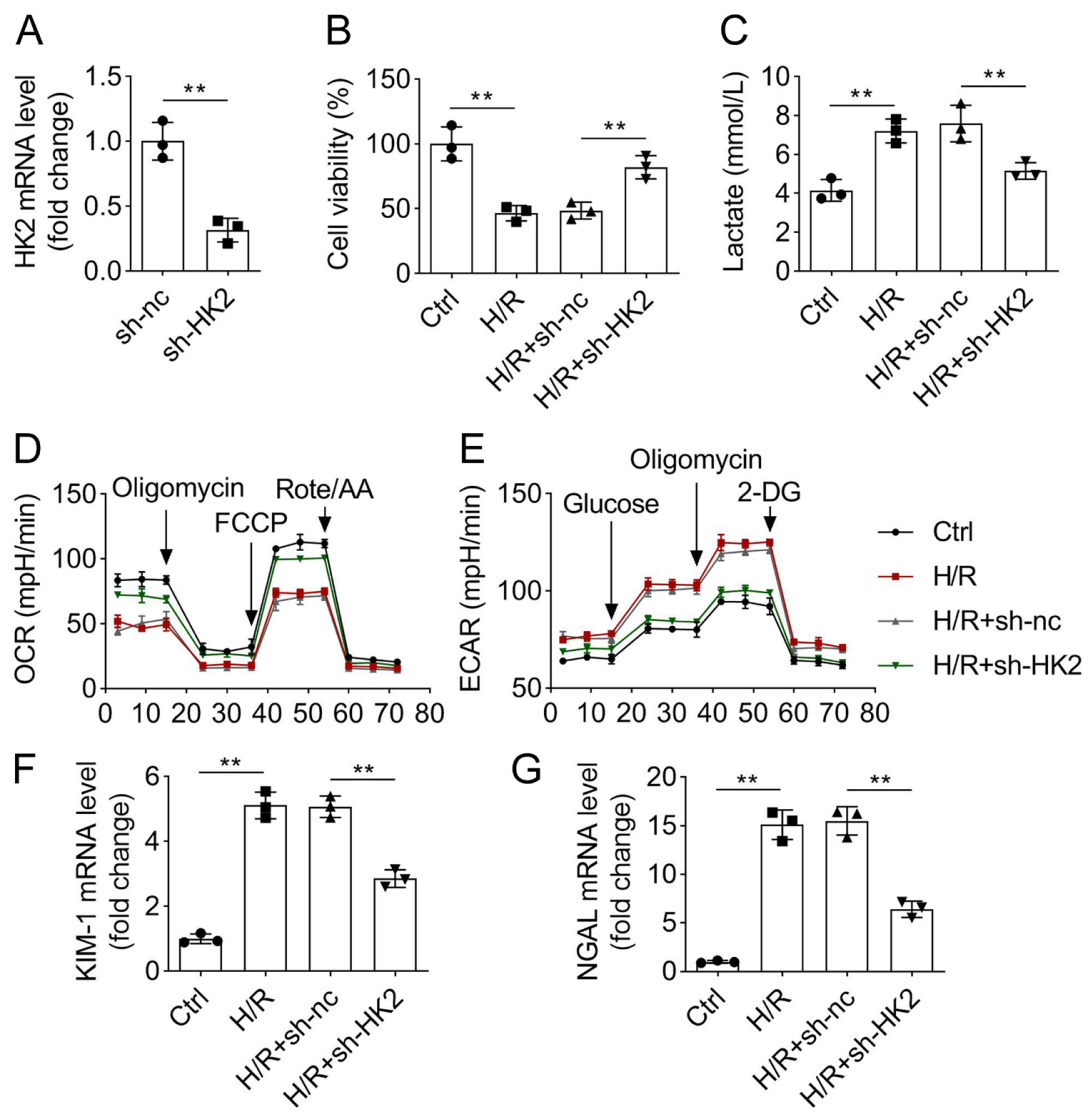
Silencing of HK2 suppresses glycolysis in H/R-induced HK-2 cells. After sh-NC and sh-HK2 transfection, (**A**) mRNA expression of HK2 was measured using qPCR, and (**B**) protein levels of HK2 were examined using western blotting. (**C**) Cell viability was determined using CCK-8 analysis. (**D**) Lactate levels in cells were detected using the corresponding kit. (**E**) ECAR and (**F**) OCR were analyzed using a Seahorse assay. (**F**) KIM-1 and (**G**) NGAL expression was measured by qPCR. **P < 0.01

HK2 regulates glycolysis in pathophysiological processes. To further confirm the role of HK2 in renal IRI, we used in vitro experiments to reveal its effects on glycolysis. HK-2 cells were induced by H/R to mimic I/R conditions in mice. We knocked down HK2, and the results showed that HK2 mRNA expression was decreased after sh-HK2 transfection (Fig. 4A). Then, cell viability was evaluated using CCK-8. H/R inhibited HK-2 cell viability, whereas HK2 knockdown rescued this inhibition (Fig. 4B). Moreover, glycolysis was evaluated by detecting lactate, OCR, and ECAR. We found that lactate and ECAR were increased, and OCR was decreased by H/R induction, suggesting that glycolysis capability was enhanced and respiratory capacity and ATP production were reduced. However, HK2 knockdown abrogated the effects of H/R on lactate, OCR, and ECAR (Fig. 4C-E). The levels of KIM-1 and NGAL were elevated by H/R treatment, while interference with HK2 reversed this elevation (Fig. 4F and G). In summary, interference with HK2 suppresses glycolysis in HK-2 cells caused by H/R.

### HK2 regulates lactylation of H3K18

**Fig. 5:**
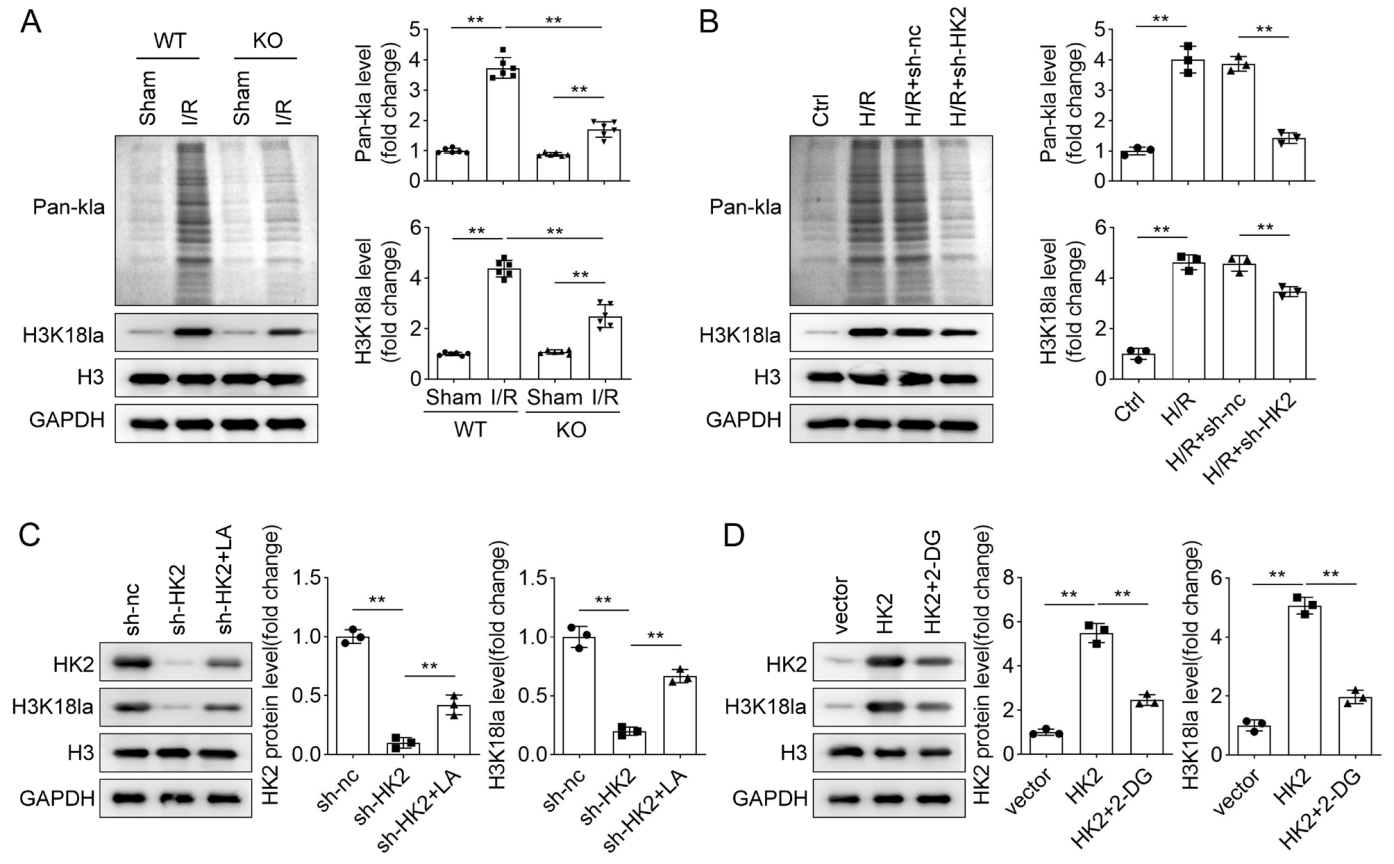
HK2 regulates lactylation of H3K18. (**A**) Western blotting was conducted to measure pan histone lactylation (pan-kla) and H3K18 lactylation (H3K18la) levels in WT and HK2-KO mice in the sham and I/R groups. H3 and GAPDH were the internal control of H3K18la and pan-kla, respectively. (**B**) Pan-kla and H3K18la levels were detected using western blotting in HK-2 cells treated with H/R and transfected with sh-HK2. (**C**) After HK2 knockdown and LA treatment, the levels of HK2 and H3K18la were measured using western blotting. (**D**) After HK2 overexpression and 2-DG treatment, the levels of HK2 and H3K18la were examined using western blotting. **P < 0.01

Under hypoxic conditions, glucose produces ATP and lactate through glycolysis. Growing evidence has reported that lactate functions as an epigenetic regulator that modifies histone lactylation [27]. Therefore, we focused on histone lactylation in this study. Here, the total lysine lactylation levels (pan-kla) and lactylation levels of H3K18 (H3K18la) were measured using western blotting. The results indicated that I/R elevated pan-kla and H3K18la in mice, which were higher in WT I/R mice than that in HK2-KO mice (Fig. 5A). Similarly, H/R increased pan-kla and H3K18la in HK-2 cells (Fig. 5B). Next, whether HK2 affected lactylation was identified. Knockdown of HK2 suppressed pan-kla and H3K18la in H/R-treated cells (Fig. 5B). Moreover, LA treatment reversed the reduction of H3K18la caused by HK2 knockdown (Fig. 5C). Overexpression of HK2 increased H3K18la, whereas 2-DG treatment counteracted this increase (Fig. 5D). The data suggested that IRI promotes H3K18 lactylation by upregulating HK2.

### H3K18 lactylation in promoter increases HK2 expression

**Fig. 6:**
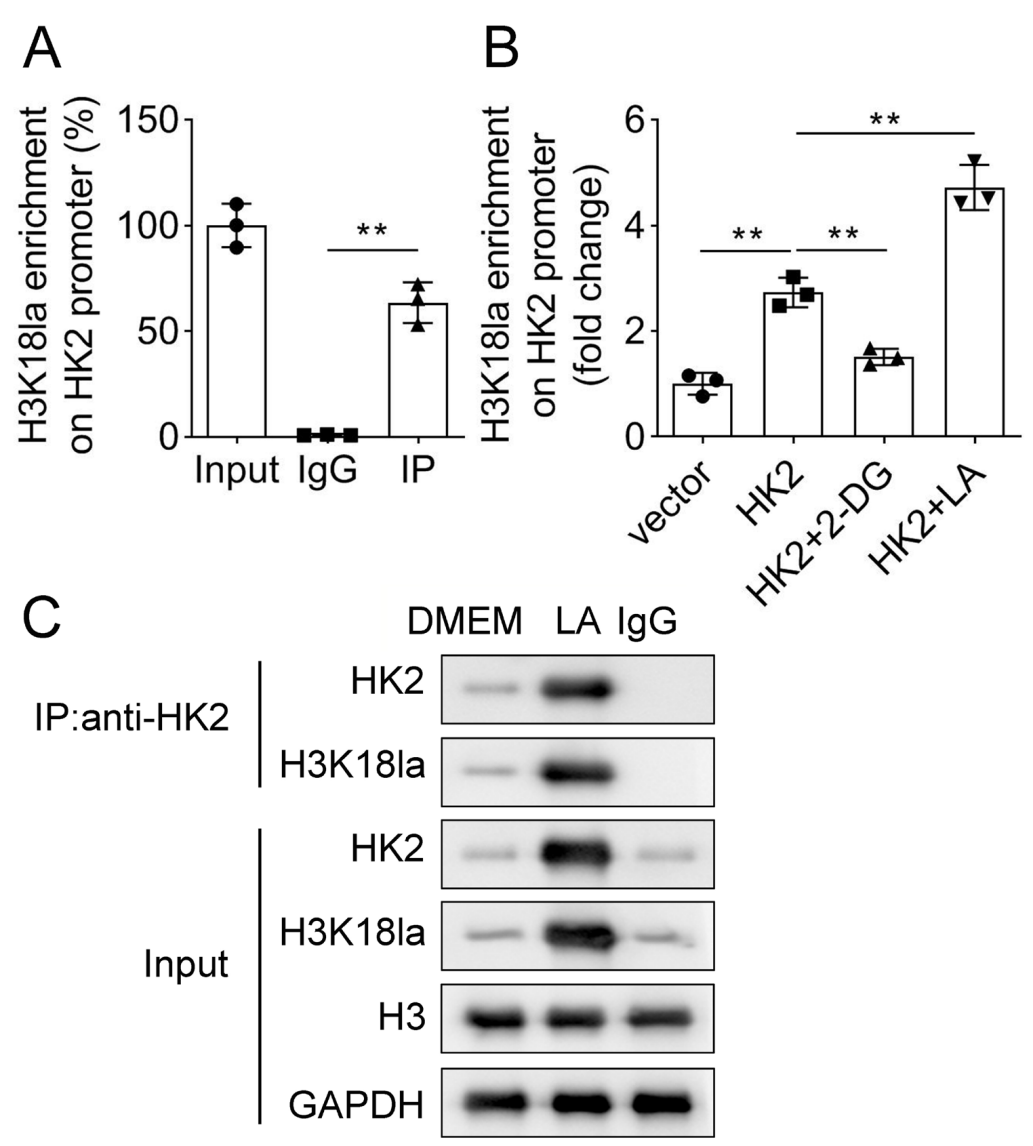
H3K18 lactylation in promoter increases HK2 expression. (**A**) The relationship between H3K18 lactylation and HK2 was evaluated using CHIP assay. (**B**) After overexpressing HK2 and treating with 2-DG or LA, H3K18la and HK2 relationship was verified using CHIP. (**C**) Following LA treatment, HK2 was immunoprecipitated, and HK2 protein and H3K18la were measured using western blotting. **P < 0.01

A previous study has indicated that H3K18 lactylation is a label for active promoters and enhancers [28]. We found that HK2 promotes lactylation of H3K18. Hence, CHIP was used to assess the association of H3K18la with HK2 promoter. The results showed that H3K18 was enriched in the HK2 promoter (Fig. 6A). Moreover, overexpression of HK2 promoted H3K18 lactylation in its promoter region. 2-DG treatment reversed H3K18la enrichment, whereas LA further increased H3K18la enrichment (Fig. 6B). Importantly, LA upregulated HK2 protein and H3K18 la levels (Fig. 6B). In short, H3K18 lactylation is enriched in HK2 promoter and then upregulated HK2 levels.

### LA reverses renal IRI alleviated by HK2 deficiency

**Fig. 7:**
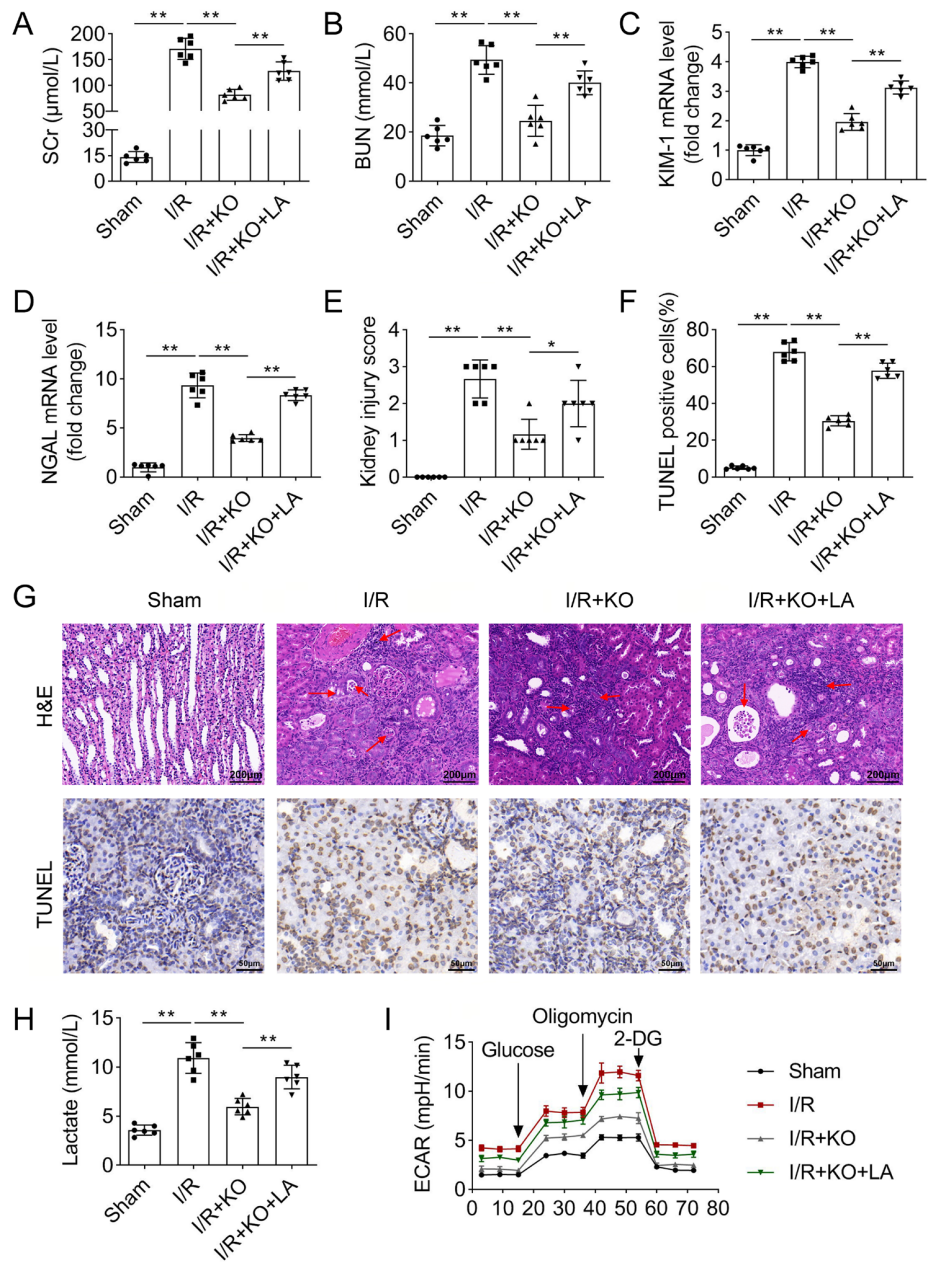
LA reverses renal IRI alleviated by HK2 deficiency. The levels of (**A**) SCr and (**B**) BUN levels in the serum of mice were detected using commercial kits. (**C**) KIM-1 and (**D**) NGAL mRNA levels were detected by qPCR in the kidney tissues. (**E**) Kidney injuey score was evaluated according to H&E staining results. (**F**) The percentage of TUNEL positive cells was quantified according to TUNEL assay results. (**G**) Representative images of H&E staining (scale bar: 200 μm; red arrows: injured tissue) and TUNEL assays (scale bar: 50 μm). (H) Lactate levels were measured using a kit. (**I**) ECAR in platelets of mice was measured using a Seahorse analysis. **P < 0.01. *P < 0.05

Finally, because lactic acid promotes H3K18 lactylation and upregulates HK2 expression, we evaluated the effects of LA on renal IRI. HK2 knockout decreased SCr and BUN levels after I/R treatment, whereas LA abrogated the decrease of their levels (Fig. 7A and B). In I/R mice, KIM-1 and NGAL mRNA levels were upregulated, and HK2 knockout decreased their levels, which was rescued by LA treatment (Fig. 7C and D). Then, reduced kidney pathology damage and cell death in I/R-induced HK2-KO mice were counteracted by LA treatment (Fig. 7E-G). Moreover, LA increased lactate levels and platelet ECAR in HK2-KO mice induced by I/R (Fig. 7H and I ). Therefore, we considered that LA increases HK2 levels by promoting H3K18 lactylation to facilitate renal IRI.

## Discussion

AKI is still a public health burden that is associated with adverse cardiovascular events and long-term CKD. Due to the heterogeneity and unknown etiology of AKI, there is no unified clinical management and treatment strategy [29]. Therefore, effective treatments for AKI need to be identified. Renal ischemia/reperfusion is a major cause of AKI induction. Inhibition of renal IRI may contribute to preventing the occurrence and development of AKI.

AST-120 has been clarified to protect the kidney from damage in CKD [7, 8]. Moreover, AST 120 can decelerate the progression of AKI to CKD [30]. Recently, it has been reported that AST-120 plays a crucial role in renal IRI. Shen et al. [11] have revealed that AST-120 treatment attenuates renal IRI, and improves cardiac function caused by I/R induction. The study of Saito et al. also revealed that AST-120 can reduce I/R-induced kidney injury [10]. However, Nakagawa et al. considered that AST-120 does not improve kidney dysfunction and does not restore damaged blood vessels caused by I/R [31]. These different conclusions may be due to the different treatment times and methods of ischemia and reperfusion. In the present study, we revealed that I/R increased SCr and BUN levels, which are markers of renal impairment [32]. Moreover, I/R promoted apoptosis and histopathology, suggesting the I/R mouse model was successfully established. After AST-120 oral administration, the levels of SCr and BUN were reduced, apoptosis was inhibited and pathology was improved. Taken together, AST-120 alleviates renal IRI in vivo.

Hypoxia is a hallmark in IRI [33]. After ischemia, blood flow is significantly reduced, and the delivery of oxygen is also reduced [24]. Although reperfusion restores total renal blood flow, local blood is altered. In particular, the outer medullary or corticomedullary junction region had only 10% of normal blood flow [34]. Thus, the outer medullary turns respiration into glycolysis to produce ATP [24]. In healthy individuals, glucose is maintained at a homeostasis level, and the kidney is considered an organ that contributes to glucose homeostasis [35]. However, abnormal glucose metabolism is involved in the development of AKI. Several glycolytic products are identified to affect kidney function. For instance, fructose 1,6-diphosphate, a glycolytic intermediate, alleviates ischemia-induced renal failure by inhibiting oxidative stress [36]. Additionally, pyruvate reduces renal impairment caused by IRI and the severity of AKI [37]. Herein, we found that HK2 was highly expressed after IRI, consistent with a previous study [17]. Moreover, HK2 expression could be affected by AST-120, speculating that HK2 may be mediated by AST-120 to influence renal IRI. The results demonstrated that AST-120 did not affect renal IRI and glycolysis capability when HK2 was knocked out, suggesting HK2 is essential in AST-120-regulated kidney protection, possible through glycolysis. Meantime, we used in vitro experiments and demonstrated that knockdown of HK2 inhibited H/R-induced glycolysis. The findings suggest that AST-120 inhibits HK-2-mediated glycolysis of proximal tubular cells to alleviate renal IRI.

Glycolysis produces lactate, which regulates histone lactation, so it is logical that we further explored histone lactation in renal IRI. H3K18 lactylation levels were elevated in I/R-induced mice and H/R-induced cells, and knockdown of HK2 reversed H3K18 lactylation levels, suggesting that HK2 facilitates glycolysis to produce lactate and thereby promotes lactylation. Histone lactylation regulates the development of diseases by affecting cellular processes. Lactylation of H3K18 inhibits Th17 cell pathogenicity to inhibit intestinal inflammation [38]. In addition, GCN5-mediated H3K18 lactylation promotes cardiac repair after myocardial infarction by anti-inflammation and pro-angiogenesis [39]. Besides, H3K18 lactylation is enriched in the tumor microenvironment, which upregulates METTL3 to facilitate tumor growth in colorectal cancer [40]. Histone lactylation transcriptional regulates HMGB1 to affect cell pyroptosis, thus promoting cerebral IRI [41]. Nevertheless, whether H3K18 lactylation is involved in renal IRI remains unknown. In the current study, lactate treatment reversed the inhibition of IRI. The results demonstrate that glycolysis produces lactate which promotes H3K18 lactylation, leading to renal IRI. Moreover, H3K18 lactylation indicates the activity of gene promoters, which can promote the transcription of downstream genes [39, 42]. We identified that H3K18 lactylation transcriptionally activated HK2, suggesting that H3K18 lactylation in turn promotes glycolysis.

In conclusion, AST-120 alleviates renal IRI by downregulating HK2 expression. Mechanically, HK2 promotes glycolysis, increases the production of lactate to promote H3K18 lactylation, and further upregulates the expression of HK2, forming a positive feedback loop. The findings provide a novel molecular mechanism for AST-120 to protect against kidney injury and provide theoretical support for its use in AKI therapy.

## Data Availability

The datasets used and/or analyzed during the current study are available from the corresponding author on reasonable request.
